# Structural Mapping of Disease-Level Community-Based Care Patterns in Rural Clinics on Remote Islands in Japan: A Questionnaire Survey

**DOI:** 10.3390/healthcare14121799

**Published:** 2026-06-22

**Authors:** Daisuke Matsubara, Kazuhiko Kotani

**Affiliations:** Center for Community Medicine, Jichi Medical University, Shimotsuke 329-0498, Japan; 99081dm@jichi.ac.jp

**Keywords:** care patterns, clinics on remote islands, community-completable conditions, contextual factors, disease-related care structures

## Abstract

**Highlights:**

**What are the main findings?**

- Community-based care patterns across 167 diseases on remote islands formed a continuous spectrum ranging from community-completable to specialist-dependent conditions.
- Community-based care patterns appeared to vary according to deployment duration and specialty composition.

**What are the implications of the main findings?**

- Community-based care patterns appeared to reflect differences in disease-related care structures and contextual factors.
- The proposed framework may support future discussions regarding education, workforce planning, and healthcare systems in remote island settings in Japan.

**Abstract:**

**Background/Objectives**: Remote islands in Japan constitute a unique medical environment in which physicians often manage a broad spectrum of clinical conditions. However, physicians practicing on remote islands have diverse medical backgrounds, and disease-level community-based care patterns in these settings have not been systematically described. This study aimed to characterize community-based care patterns across diseases in clinics on remote islands in Japan using an exploratory conceptual framework and to examine whether facility- and physician-related attributes were associated with these patterns. **Methods**: We conducted a questionnaire survey in February 2023 involving rural clinics on remote islands in Japan. For each disease, respondents reported community involvement at three clinical stages—initial consultation, follow-up, and completion of care—yielding eight possible care patterns (000–111). Primary community completeness was defined as the proportion of clinics reporting community-based involvement in initial consultation and completion of care (P111 + P101). Diseases were ranked according to this metric and stratified into three predefined conceptual zones (upper, middle, and lower). Subgroup analyses examined differences in primary community completeness according to facility- and physician-related attributes, including deployment duration, prior rural practice experience, career length, and specialty composition. **Results**: We analyzed data from 23 clinics covering 167 diseases. Diseases formed a continuous gradient ranging from community-completable to specialist-dependent conditions. Differences in community-based care patterns were most pronounced in the middle zone. Deployment duration was associated with directional differences in community-based care patterns, whereas specialty composition was associated with larger subgroup differences. In contrast, diseases in the lower zone demonstrated relatively stable specialist-dependent patterns regardless of facility- or physician-related attributes. **Conclusions**: This exploratory study proposed a conceptual framework for characterizing community-based care patterns across diseases in clinics on remote islands in Japan. The findings suggest that community-based care patterns on remote islands may reflect differences in disease-related care structures as well as contextual factors. The proposed framework may support future discussions regarding education, workforce planning, and healthcare systems in remote island settings in Japan.

## 1. Introduction

The shortage of healthcare resources and physicians on remote islands remains a longstanding challenge [1,2,3]. On many remote islands in Japan, healthcare is primarily delivered through small community-based clinics that often collaborate closely with referral hospitals on the mainland to provide advanced and specialist care [1,2,3]. These clinics are typically staffed by a limited number of healthcare professionals, primarily physicians and nurses, and have limited access to onsite specialist services. In these settings, physicians often practice independently and provide comprehensive care across a broad spectrum of conditions [4,5]. Such environments require a generalist or primary care approach rather than specialist-focused practice. A systematic review indicated that physicians in rural settings, including remote island clinics, must manage a diverse range of clinical problems extending beyond the traditional boundaries of internal medicine [6].

Japan faces a super-aging society characterized by rapid demographic changes driven by a declining birth rate and a growing elderly population. These demographic changes are particularly pronounced on remote islands, where the proportion of elderly residents exceeds the national average (37.1% vs. 26.9%) [7]. An aging population alters the disease burden by increasing the prevalence of chronic and complex conditions [6], thereby amplifying the importance of community-based and longitudinal care in these settings. However, physicians practicing on remote islands have diverse backgrounds, including differences in deployment duration, prior rural experience, career length, and specialty composition, which may influence the scope of care provided [2,5]. Previous studies have described the broad and context-dependent nature of rural and remote medical practice [4,5,6] but have primarily focused on physician roles and the general scope of practice rather than disease-specific patterns of community-based involvement. Consequently, it remains unclear which conditions can be managed within community-based clinics and which tend to require specialist-dependent care pathways in remote island settings. Under these circumstances, understanding disease-level community-based care patterns may inform workforce planning, training, and the design of locally appropriate healthcare systems.

Therefore, this study aimed to characterize disease-level community-based care patterns across remote island clinics using an exploratory conceptual framework. Furthermore, we examined whether facility- and physician-related attributes were associated with these care patterns.

## 2. Materials and Methods

### 2.1. Study Design and Participants

This cross-sectional questionnaire survey was conducted in February 2023. Eligible facilities included rural clinics on remote islands across Japan that were staffed by full-time physicians providing community-based care. Clinics with incomplete questionnaire data were excluded from the final analysis. Participants were recruited through a two-step process targeting rural clinics on remote islands in Japan. Initial study invitations were broadly distributed to 224 eligible facilities, and clinics that provided informed consent subsequently received detailed study information and questionnaires for second-stage enrollment. All clinics that proceeded to the second-stage enrollment completed the survey. Questionnaires were distributed and collected via mail. The survey collected data on clinic characteristics and physician attributes, including years of clinical experience, duration of remote island practice, prior rural practice experience, and specialty background (internal medicine, surgery, or primary care). A total of 168 diseases were selected through discussions among physicians experienced in remote medicine to cover a broad spectrum ranging from common primary care conditions (e.g., acute upper respiratory infection and allergic rhinitis) to highly specialized disorders (e.g., acute leukemia and malignancies).

### 2.2. Disease Classification and Conceptual Framework

#### 2.2.1. Eight Possible Binary Care Patterns

For each disease, respondents indicated their clinic’s involvement at three clinical stages: initial consultation, follow-up, and completion of care (1 = involved, 0 = not involved). This process yielded eight possible binary care patterns (000–111) for each disease (Supplementary Materials Table S1).

Based on these care patterns, we developed an exploratory conceptual framework describing community-based care patterns across diseases (Table 1).

#### 2.2.2. Primary and Secondary Classification

For the primary classification, which focuses on acute care patterns and referral structure, diseases were categorized as follows:

- **Community-completable diseases**: Conditions in which the initial consultation and completion of care were managed within community-based clinics (patterns 111 and 101). Patterns 111 and 101 were combined because they shared two core structural features: community-based initial care and completion of care within community settings.
- **Quasi-community-completable diseases**: Conditions in which initial consultation and follow-up care were managed locally, whereas completion of care required referral to specialist centers (pattern 110).
- **Specialist-dependent diseases**: Conditions requiring specialist-led care from the outset, with minimal community involvement beyond initial triage (patterns 000 and 100).

For the secondary classification, which focuses on chronic and longitudinal phases of care, diseases were categorized into:

- **Shared/step-down care diseases:** Conditions in which follow-up and/or completion of care occurred in the community following specialist-led initial management (patterns 010 and 011).
- **Terminal/follow-up care diseases**: Conditions in which community-based clinics primarily provided end-of-life or long-term follow-up care (pattern 001).

To assess the robustness of these conceptual categories, specific patterns were examined to determine whether they emerged as distinct groups using a 60% agreement threshold across clinics. This threshold was used solely for exploratory structural assessment rather than category redefinition. Although pattern 100 (community triage only) may conceptually differ from pattern 000 (no community involvement), pattern 100 did not demonstrate a distinct structural predominance across diseases in the present dataset. Therefore, pattern 100 was grouped with pattern 000 under specialist-dependent diseases for exploratory interpretation and visualization. No threshold-based reassignment was applied to the chronic-phase categories.

### 2.3. Structural Metrics

#### 2.3.1. Primary Community Completeness and Three Predefined Conceptual Zones

For each disease, primary community completeness was quantified as the combined proportion of clinics reporting community-based involvement at the initial consultation and completion stages, defined as

Primary community completeness = P(111) + P(101)

where P(111) and P(101) represent the proportions of facilities reporting the corresponding care patterns.

To facilitate interpretation, diseases were stratified into three predefined conceptual zones based on primary community completeness:

- **Upper zone (≥0.60)**: Diseases predominantly managed within community-based clinics.
- **Middle zone (0.30–0.59)**: Diseases that fall between the upper and lower zones.
- **Lower zone (<0.30)**: Diseases requiring specialist-dependent care.

These thresholds were fixed a priori and applied uniformly across all analyses.

#### 2.3.2. Secondary Dominance

Acute–chronic structural differentiation was assessed by calculating secondary dominance, defined as the difference between shared-care patterns (010 and 011) and terminal-care patterns (001):

Secondary dominance = P(010) + P(011) − P(001).

### 2.4. Outcomes

The primary outcome was the exploratory characterization of reported community-based care patterns across diseases in remote island clinics, represented by primary community completeness (P(111) + P(101)). This metric was intended to describe the extent of reported community-based involvement in initial consultation and completion of care for specific diseases and to provide a conceptual spectrum ranging from community-completable to specialist-dependent conditions. The metric was designed to evaluate care structure rather than disease severity, clinical complexity, or patient outcomes.

Secondary outcomes included (1) assessment of acute–chronic structural differentiation and (2) subgroup evaluation of differences in primary community completeness based on facility- and physician-related attributes, including deployment duration, prior rural practice experience, career length, and specialty composition.

### 2.5. Statistical Analysis

For each disease, proportions of the eight care patterns (000–111) were calculated across participating clinics. Diseases were then sorted in descending order of primary community completeness. This ordering was applied consistently across all figures to ensure comparability across analyses. As a sensitivity analysis, alternative predefined thresholds (upper ≧ 0.65 and lower < 0.25) were additionally applied to assess whether the overall zone-based interpretation was robust to changes in threshold definition. In addition, bootstrap 95% confidence intervals for zone-specific medians were estimated using non-parametric resampling with 10,000 iterations.

#### Subgroup Analyses

Subgroup analyses were performed for three exposure-type attributes—deployment duration (median split), prior rural practice experience (binary), and career length (median split)—and one compositional attribute (specialty: internal medicine, surgery, or primary care). Subgroup-specific primary community completeness was calculated for each disease.

For exposure-type attributes (deployment duration, prior rural practice experience, and career length), the directional difference between subgroups was defined as

ΔPrimary = (P111 + P101)_higher/exposed − (P111 + P101)_lower/non-exposed.

For specialty composition, structural divergence was quantified as

RangePrimary = max(P111 + P101)_specialty − min(P111 + P101)_specialty.

Differences in ΔPrimary and RangePrimary across the predefined primary completeness zones (upper, middle, lower) were examined exploratorily using Kruskal–Wallis tests for non-parametric comparisons. Because these analyses were exploratory and disease-level observations were not fully independent, subgroup comparisons were intended as descriptive structural assessments rather than formal inferential hypothesis testing, and adjustments for multiple comparisons were not performed. Absolute differences, |ΔPrimary|, were calculated to assess dispersion independent of direction.

All analyses and figure generation were performed using MATLAB (version R2020a, MathWorks, Natick, MA, USA). To ensure reproducibility and consistent ranking across figures, data preprocessing, disease ranking, subgroup analyses, and figure construction were implemented using custom-built scripts. Statistical comparisons were exploratory and intended to support structural pattern interpretation rather than formal inferential evaluation.

## 3. Results

Of the twenty-four clinics participating in this study, one was excluded due to incomplete information. Cardiopulmonary arrest was also excluded from the initial 168 diseases because it lacked a complete three-stage structure. Consequently, the final analysis included 23 clinics covering 167 diseases. Participating physicians represented diverse clinical backgrounds with respect to deployment duration, prior rural practice experience, career length, and specialty composition. Physicians reported a median career length of 9.0 years (interquartile range [IQR]: 7.0–32.0) and a median remote island practice duration of 2.0 years (IQR: 1.0–12.0). Nine clinics reported prior rural practice experience, whereas two had missing data for this variable. Specialty backgrounds included internal medicine (*n* = 7), surgery (*n* = 7), and primary care (*n* = 9).

**Figure 1:**
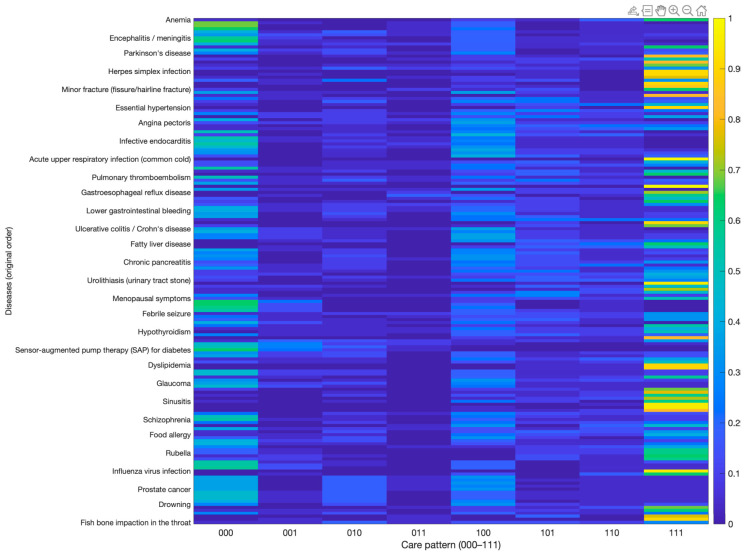
Heatmap of disease-level distribution across eight care patterns. Each row represents a disease, and each column represents one of the eight binary care patterns (000–111), defined by community-based involvement across three clinical stages: initial consultation, follow-up, and completion of care (1 = presence of community-based involvement, 0 = absence). Color intensity indicates the proportion of clinics reporting each pattern. Representative disease labels are shown for readability. This figure visualizes the distribution of care patterns across diseases and informs the exploratory conceptual classification presented in Table 1. Distinct horizontal color patterns reflect heterogeneity in community-based care structures among diseases.

Figure 1 illustrates the distribution of diseases across the eight care patterns in the original survey order, showing 30 representative disease labels. Visual distribution patterns were observed across diseases, including conditions with higher proportions of community-based patterns (111 and 101), specialist-dependent patterns (000 and 100), and patterns involving longitudinal community participation (011, 101, and 001). These distributions informed the exploratory conceptual framework presented in Table 1.

**Figure 2:**
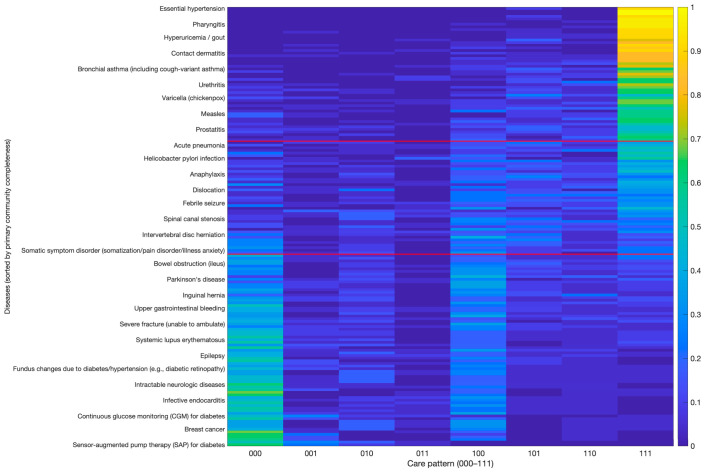
Heatmap of diseases ranked by primary community completeness. Diseases were ordered according to primary community completeness, defined as the combined proportion of clinics reporting community-based involvement in initial consultation and completion of care (P111 + P101). The heatmap color scale represents the proportion of each individual care pattern (000–111). Horizontal red lines indicate predefined conceptual thresholds (≥0.6, 0.3–0.6, and <0.3), corresponding to the upper, middle, and lower zones used for exploratory structural interpretation. Representative disease labels are shown for readability. The ordered heatmap allows visualization of gradual transitions in care-pattern distributions according to increasing primary community completeness. Supplementary Materials Table S2 provides detailed values.

Figure 2 presents diseases ranked by primary community completeness (P111 + P101), with diseases distributed along a continuous spectrum. Using predefined conceptual thresholds (indicated by red lines: ≧0.6, 0.3–0.6, and <0.3), diseases were stratified into upper, middle, and lower zones for exploratory structural interpretation. Diseases in the upper zone (e.g., essential hypertension and pharyngitis) were more frequently associated with reported community-based involvement, whereas diseases in the lower zone (e.g., severe fracture and malignancies) were more consistently associated with reported specialist-dependent care patterns. Diseases in the middle zone (e.g., acute pneumonia and dislocation) demonstrated intermediate distributions across clinics. Supplementary Materials Table S2 provides the exact order of diseases and corresponding values.

**Figure 3:**
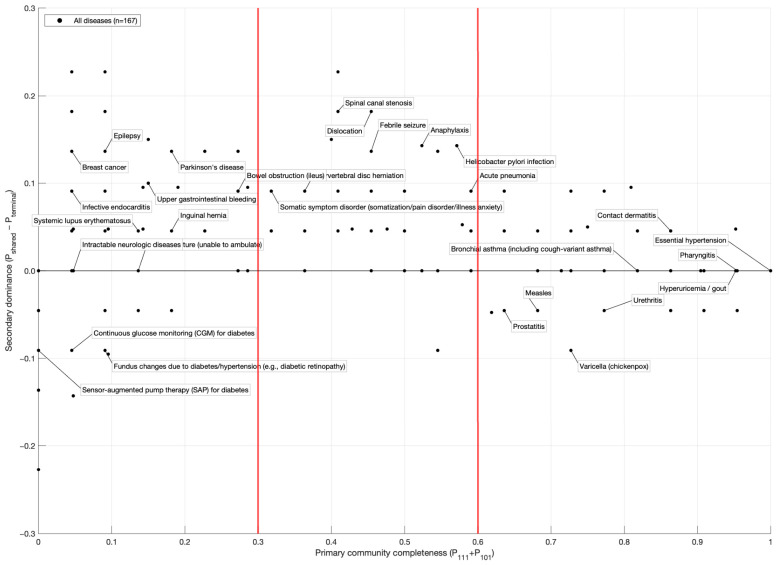
Secondary dominance versus primary community completeness. The x-axis represents primary community completeness (P111 + P101). Vertical red lines indicate the same predefined conceptual thresholds used in Figure 2. The y-axis represents secondary dominance, calculated as the difference between the shared or step-down care component and the terminal or follow-up care component (P011 + P010–P001). Positive values indicate relatively greater representation of shared or step-down care patterns following specialist-led initiation, whereas negative values indicate relatively greater representation of terminal or follow-up-oriented community involvement. The distribution along the y-axis suggests heterogeneity in longitudinal community involvement among diseases with similar levels of primary community completeness. Supplementary Materials Table S3 provides detailed values.

Figure 3 illustrates the relationship between primary community completeness (111 and 101) and secondary care dominance (010 + 011–001) across 167 diseases. Even among diseases with similar levels of primary community completeness, patterns of longitudinal community involvement differed across diseases, particularly in the relative representation of shared/step-down care and terminal/follow-up-related community involvement (Supplementary Materials Table S3).

**Figure 4:**
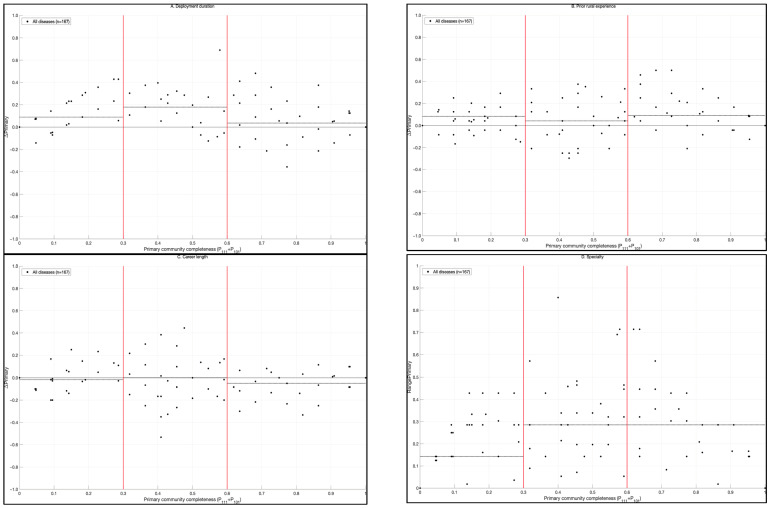
Differences in primary community completeness according to facility- and physician-related attributes. Diseases were ordered identically to Figure 2 and Figure 3 according to overall primary community completeness (P111 + P101). Vertical red lines indicate the predefined conceptual thresholds for the upper, middle, and lower zones. Panels (**A**–**C**) show subgroup differences ((**A**): deployment duration; (**B**): prior rural practice experience; (**C**): career length) in primary community completeness (ΔPrimary), calculated as the subgroup-specific value of (P111 + P101) minus that of the reference group. Positive values indicate higher primary completeness in the exposed or higher-experience group. Dotted horizontal lines indicate median values within each zone. Panel (**D**) shows subgroup differences according to specialty composition using RangePrimary, defined as the difference between the maximum and minimum specialty-specific values of (P111 + P101) across internal medicine, surgery, and primary care groups. Supplementary Materials Tables S4 and S5 provide detailed values and statistical comparisons.

Figure 4 illustrates exploratory subgroup-related distributional patterns in primary community completeness according to facility- and physician-related attributes. Regarding deployment duration (Figure 4A), directional differences in ΔPrimary appeared more pronounced in the middle zone than in the upper or lower zones (median ΔPrimary: upper/middle/lower: 0.036, 0.179, 0.089). Prior rural practice experience (Figure 4B) showed heterogeneous distributional patterns without a consistent directional tendency (median ΔPrimary: upper/middle/lower: 0.091, 0.042, 0.083). Career length (Figure 4C) showed smaller overall directional differences relative to deployment duration and prior rural experience (median ΔPrimary: upper/middle/lower: −0.050, 0, −0.017). Across Figure 4A–C, absolute differences (|ΔPrimary|) tended to be greatest in the middle zone relative to the upper or lower zones (Supplementary Materials Table S4). Exploratory Kruskal–Wallis comparisons are presented in Supplementary Materials Table S4.

Finally, specialty-related subgroup variability (Figure 4D) appeared greater in the middle and upper zones (median RangePrimary: upper/middle/lower: 0.286, 0.286, 0.143) (Supplementary Materials Tables S4 and S5). Similar zone-based tendencies were observed in sensitivity analysis using alternative predefined thresholds (upper ≧ 0.65 and lower < 0.25), with subgroup-related variability remaining most pronounced in the middle zone (Supplementary Materials Table S6). Bootstrap 95% confidence intervals showed broadly similar zone-specific distributional patterns (Supplementary Materials Table S4).

## 4. Discussion

This exploratory study proposed a conceptual framework for describing disease-level community care patterns across clinics on remote islands in Japan, based on primary community completeness. The findings suggested a continuous spectrum of community-based involvement in the acute-phase care setting, ranging from community-completable to specialist-dependent care patterns. Incorporating secondary dominance further indicated that diseases with similar levels of primary community completeness may differ in their patterns of chronic and longitudinal community involvement. Moreover, facility- and physician-related attributes were associated with community care patterns, particularly for diseases in the middle zone. Together, these findings suggest that community care patterns on remote islands may reflect differences in disease-related care structures, physician practice patterns, and contextual factors.

Owing to their unique medical environment, physicians on remote islands tend to provide comprehensive care across a broad spectrum of conditions [4]. A systematic review examining patients’ reasons for encounter in primary care settings reported that “non-internal medicine-related reasons” accounted for 4% to 40% of visits, regardless of population density [6], a finding conceptually consistent with our observation that many diseases in the upper zone were associated with broad community-based involvement. These included common acute infections (e.g., acute respiratory infection and acute gastroenteritis), chronic lifestyle-related conditions (e.g., essential hypertension, type 2 diabetes, and osteoporosis), mild dermatologic/allergic disorders (e.g., atopic dermatitis and herpes zoster), minor trauma (e.g., minor fracture, fishhook injury, animal bite, and epistaxis), and functional disorders (e.g., functional gastrointestinal disorder, migraine, Ménière’s disease, and menopausal symptoms). These conditions can often be diagnosed and managed using relatively standardized approaches, require fewer specialist procedures, and are more frequently managed within community-based clinics regardless of facility- or physician-related attributes [5].

Facility- and physician-related attributes were associated with community care patterns on remote islands, particularly for diseases in the middle zone. Deployment duration was associated with directional differences, whereas specialty composition was associated with greater subgroup differences. Absolute subgroup differences were most pronounced in the middle zone, indicating greater contextual variability in this domain. Diseases in the middle zone included (1) skill-expandable acute conditions (e.g., acute pneumonia, acute pyelonephritis, and acute kidney injury); (2) chronic diseases requiring advanced but developable management capacity (e.g., chronic heart failure, chronic kidney disease, diabetes requiring insulin initiation, rheumatoid arthritis, chronic hepatitis, and hypo/hyperthyroidism); (3) procedure-dependent conditions sensitive to specialty composition (e.g., dislocation, fish bone impaction, hemorrhoids, and gastric anisakiasis); (4) intermediate emergency conditions (e.g., paroxysmal supraventricular tachycardia, anaphylaxis, and febrile seizure); (5) diagnostic-resource-sensitive conditions (e.g., secondary hypertension and sleep apnea syndrome); and (6) longitudinal psychiatric conditions (e.g., dementia and somatic symptom disorder). These conditions were more sensitive to differences in facility capacity, physician experience, and specialty composition. Previous studies have suggested that rural and remote healthcare capacity is influenced by physician training, local resources, and system-level support [8,9,10]. Our findings extend these observations by suggesting that such contextual variability may differ according to disease category and may be pronounced among diseases in the middle zone.

Conversely, diseases in the lower zone demonstrated relatively stable, specialist-dependent care patterns regardless of facility- or physician-related factors. These included conditions requiring chemotherapy (e.g., acute leukemia and malignancies), intensive care (e.g., acute heart failure and acute pancreatitis), urgent surgical intervention (e.g., acute appendicitis, subdural hematoma, and severe fractures), obstetric management (e.g., childbirth and ectopic pregnancy), psychiatric or neurologic management (e.g., schizophrenia, Parkinson’s disease, and intractable neurologic disorders), and disease-specific procedures (e.g., intussusception). These findings are broadly consistent with previous reports describing reliance on mainland specialist referral systems for acute and procedure-intensive conditions [3,11,12,13]. Our findings further suggest that even for specialist-dependent diseases, community-based clinics may continue to play important roles during chronic and longitudinal phases of care [14].

Medical care constitutes a fundamental component of social infrastructure. Regardless of residence, access to trustworthy medical professionals and reliable services remains essential [15]. However, the concept of “ideal” medical care varies depending on community characteristics. A qualitative study involving residents across four Japanese community types—metropolitan (*n* = 22), provincial (*n* = 25), mountain/fishing villages (*n* = 31), and remote islands (*n* = 27)—demonstrated that rural residents emphasized lifestyle-oriented care, such as “locally appropriate standards,” whereas urban residents prioritized highly specialized services [16]. This suggests that residents recognize and adapt their expectations to the specific characteristics of their communities. Given the intrinsic link between medical care and quality of life [17], our findings provide an exploratory structural perspective for understanding community-based care patterns in remote island settings. By identifying areas showing greater contextual variability—particularly within the middle zone—the proposed framework may contribute to future educational and policy discussions on locally appropriate care systems on remote islands. Similar challenges related to healthcare access, workforce distribution, and reliance on generalist practice have also been reported in geographically isolated settings internationally, including rural and remote regions in Australia and other island communities [9,10,12]. Although healthcare systems differ substantially across countries, the present exploratory framework may provide a conceptual reference for future studies examining community-based care patterns in other remote or resource-limited settings.

This study has several limitations. First, the relatively small number of participating clinics and the recruitment strategy may limit the generalizability and representativeness of the findings and introduce selection bias. In addition, the reported care patterns were based on physician self-report and were not independently verified against actual clinical practice patterns. Second, although we included a broad range of conditions, the selected diseases and conceptual classification were based on expert consensus without formal validation. Therefore, the proposed framework should be interpreted as exploratory and hypothesis-generating. Third, the binary classification of care involvement (0/1) across three stages may oversimplify actual clinical practice and may not fully capture gradations of involvement, shared-care arrangements, or dynamic referral processes. Similarly, primary community completeness and secondary dominance represent simplified exploratory structural metrics and may not fully distinguish clinically different forms of community involvement. In addition, the predefined zonal thresholds were intended for exploratory structural interpretation rather than definitive cutoffs, although sensitivity analysis using alternative predefined thresholds demonstrated similar overall zone-based tendencies. Fourth, subgroup findings should be interpreted cautiously, as differences between groups may reflect instability in estimates arising from small subgroup sizes, overlapping confounding factors, physician self-selection into remote island practice, and unmeasured structural differences across clinics. In addition, disease-level observations were not fully independent because care patterns may have been influenced by shared contextual factors, referral structures, and physician practice environments across clinics and diseases. Therefore, subgroup comparisons and associated *p*-values should be interpreted as exploratory descriptive assessments rather than formal inferential testing. Similarly, although bootstrap confidence intervals were additionally estimated to assess uncertainty around zone-specific medians, these analyses should also be regarded as exploratory due to the disease-level descriptive design and limited sample size. Finally, the present framework focused on structural care patterns and did not assess patient-level outcomes, quality of life, or clinical effectiveness, which should be explored in future studies.

## 5. Conclusions

This exploratory study proposed a conceptual framework describing a continuous spectrum of community-based care patterns across diseases in clinics on remote islands in Japan. Our findings suggest that community-based care patterns may reflect differences in disease-related care structures, physician practice patterns, and contextual factors. Diseases in the upper zone tended to show greater reported community-based involvement, whereas those in the lower zone were more consistently associated with reported specialist-dependent care patterns. Diseases in the middle zone exhibited greater subgroup-related variability across clinics. In specialist-dependent conditions, community involvement appeared to be more prominent during chronic and longitudinal phases of care. Overall, the proposed framework may support future discussions regarding education, workforce planning, and healthcare systems in remote island settings in Japan.

## Figures and Tables

**Table 1:** Primary and secondary analytic categories derived from the eight binary care patterns.

Analytic Level	Analytic Category	Binary Pattern	Initial Care	Follow-Up Care	Completion of Care	Clinical Interpretation
Primary	Community-completable	111	Community	Community	Community	Diagnosis, management, and completion of care can be achieved entirely within community-based clinics.
		101	Community	Specialist	Community	Temporary specialist involvement is required, but care is ultimately completed in the community.
	Quasi-community-completable	110	Community	Community	Specialist	Community-based management with referral required for definitive treatment.
	Specialist-dependent	000	Specialist	Specialist	Specialist	Specialist involvement is required throughout all phases of care.
		100	Community	Specialist	Specialist	Community involvement is limited to initial triage or referral.
Secondary	Shared/Step-down care	011	Specialist	Community	Community	Community-based follow-up and completion after specialist-led initiation.
		010	Specialist	Community	Specialist	Shared management during the follow-up phase.
	Terminal/Follow-up care	001	Specialist	Specialist	Community	Community involvement is primarily focused on end-of-life or long-term follow-up.

## Data Availability

The data presented in this study are available from the corresponding author upon reasonable request. The data are not publicly available because of ethical restrictions related to the small number of participants and the potential risk of identifying the facilities.

## Supplementary Materials

The following supporting information can be downloaded at: https://www.mdpi.com/article/10.3390/healthcare14121799/s1: Table S1. Definitions of the eight binary care patterns. Table S2. Ordering of 167 diseases by primary community completeness and their corresponding values. Table S3. Primary community completeness and secondary care dominance for 167 diseases. Table S4. Zone-specific distributions and bootstrap 95% confidence intervals for Figure 4A–D. Table S5. Specialty-specific primary community completeness and corresponding primary range values for each disease (Figure 4D). Table S6. Sensitivity analysis of zone-specific distributions for Figure 4A–D.
