# Supplementary material for: Structural Mapping of Disease-Level Community-Based Care Patterns in Rural Clinics on Remote Islands in Japan: A Questionnaire Survey
Source: Healthcare (Basel). 2026 Jun 22;14(12):1799. doi: 10.3390/healthcare14121799 (PMC13300563; doi:10.3390/healthcare14121799)
Supplement: Supplementary file 1 [file healthcare-14-01799-s001.zip › HC-4232458 Supplementary Table S1.pdf]

Supplementary Table S1. Definition of the eight binary care patterns

Binary pattern	Initial care	Follow-up care	Completion of care	Description
000	Specialist	Specialist	Specialist	Specialist-led care throughout all phases
001	Specialist	Specialist	Community	Community involvement mainly at terminal or follow-up phase
010	Specialist	Community	Specialist	Shared management during follow-up phase
011	Specialist	Community	Community	Step-down care after specialist initiation
100	Community	Specialist	Specialist	Community involvement limited to initial triage
101	Community	Specialist	Community	Community completion with temporary specialist input
110	Community	Community	Specialist	Community-based management with referral for completion
111	Community	Community	Community	Care fully managed and completed in the community
